# Retrospective Exposure Estimation and Predicted versus Observed Serum Perfluorooctanoic Acid Concentrations for Participants in the C8 Health Project

**DOI:** 10.1289/ehp.1103729

**Published:** 2011-08-03

**Authors:** Hyeong-Moo Shin, Verónica M. Vieira, P. Barry Ryan, Kyle Steenland, Scott M. Bartell

**Affiliations:** 1 School of Social Ecology, University of California, Irvine, California, USA; 2 Department of Environmental Health, Boston University, Boston, Massachusetts, USA; 3 Department of Environmental Health, Emory University, Atlanta, Georgia, USA; 4 Program in Public Health, Department of Statistics, and Department of Epidemiology, University of California, Irvine, California, USA

**Keywords:** exposure, perfluorooctanoic acid, pharmacokinetics, serum

## Abstract

Background: People living or working in eastern Ohio and western West Virginia have been exposed to perfluorooctanoic acid (PFOA) released by DuPont Washington Works facilities.

Objectives: Our objective was to estimate historical PFOA exposures and serum concentrations experienced by 45,276 non-occupationally exposed participants in the C8 Health Project who consented to share their residential histories and a 2005–2006 serum PFOA measurement.

Methods: We estimated annual PFOA exposure rates for each individual based on predicted calibrated water concentrations and predicted air concentrations using an environmental fate and transport model, individual residential histories, and maps of public water supply networks. We coupled individual exposure estimates with a one-compartment absorption, distribution, metabolism, and excretion (ADME) model to estimate time-dependent serum concentrations.

Results: For all participants (*n* = 45,276), predicted and observed median serum concentrations in 2005–2006 are 14.2 and 24.3 ppb, respectively [Spearman’s rank correlation coefficient (*r*_s_) = 0.67]. For participants who provided daily public well water consumption rate and who had the same residence and workplace in one of six municipal water districts for 5 years before the serum sample (*n* = 1,074), predicted and observed median serum concentrations in 2005–2006 are 32.2 and 40.0 ppb, respectively (*r*_s_ = 0.82).

Conclusions: Serum PFOA concentrations predicted by linked exposure and ADME models correlated well with observed 2005–2006 human serum concentrations for C8 Health Project participants. These individualized retrospective exposure and serum estimates are being used in a variety of epidemiologic studies being conducted in this region.

Perfluorooctanoic acid (PFOA, or C8) is one of the two most studied and prevalent worldwide perfluorinated compounds, along with perfluorooctane sulfonate (PFOS). PFOA has been used in the manufacture of Teflon® and other fluoropolymers. The primary sources of PFOA to the environment are direct emissions from the manufacturing facilities to air and water, indirect emissions from landfill leaching to groundwater, and farther long-range transport via ocean current and atmospheric dispersion (McMurdo et al. 2008; Shin et al. 2011). Effluent from wastewater treatment plants may also contribute to PFOA contamination in the general environment (Loganathan et al. 2007; Sinclair and Kannan 2006). In spite of the voluntary phase-out on the use of PFOA by major manufacturing companies, the detection of PFOA in wastewater influent indicates it has been released from consumer products made of this chemical (Loganathan et al. 2007). When products that contain PFOA are used indoors, it accumulates indoors, especially in house dust (Strynar and Lindstrom 2008). Other potential sources of PFOA for human intake are assumed to be food and beverages, which are either primarily contaminated or secondarily contaminated by food packaging materials (Begley et al. 2005). In the United States, PFOA was detected in the serum of most people with a median of 4 ppb in 2003–2004, 2005–2006, and 2007–2008 [National Health and Nutrition Examination Survey (NHANES) 2011].

Although there have been some animal studies of the toxic effects of PFOA (Abdellatif et al. 1991; Andersen et al. 2008; Kennedy et al. 2004; Lau et al. 2006, 2007; Luebker et al. 2005; Nilsson et al. 1991), the health effects in human subjects are still largely unknown (Steenland et al. 2010). Three longitudinal studies reported half-life estimates for PFOA in human serum: *a*) a median of 3.5 years from a study of 28 retired workers with 5 years of follow-up (Olsen et al. 2007); *b*) a median of 2.3 years from a study of 200 people who were exposed to contaminated public water, after 1 year of follow-up (Bartell et al. 2010); and *c*) a geometric mean of 3.3 years from 138 participants (45 children, 46 mothers, and 47 men) in a German study who were also exposed via drinking water, after 2 years of follow-up (Brede et al. 2010).

Drinking water in the Mid-Ohio Valley has been presumably contaminated with PFOA released from the DuPont Washington Works facilities near Parkersburg, West Virginia, since 1951. PFOA emissions steadily increased as production of PFOA-related products increased over time, peaking in 1999 and then sharply decreasing after control strategies were implemented (DuPont 2008). PFOA emitted from the stacks was transported according to prevailing wind directions and settled to the ground surface by wet or dry deposition. Deposited PFOA infiltrated through the unsaturated zone, a region between the land surface and groundwater aquifer, with precipitation, and it eventually reached the saturated groundwater aquifer. PFOA released into the Ohio River contaminated the groundwater aquifer that interacts with the river (Shin et al. 2011).

We recently developed a more sophisticated multicompartment environmental fate and transport model to estimate retrospective year-by-year PFOA concentrations in air, groundwater, and six public water supplies involved in the C8 Health Project, a cross-sectional study conducted from 2005 to 2006 (Shin et al. 2011).

In this study, we linked retrospective air and water concentration predictions from the Shin et al. (2011) model to individual residential histories for 45,276 participants from the C8 Health Project, predicting year-by-year PFOA exposures based on their individual residential histories and likely water sources. We then linked these individual annual exposure estimates to an absorption, distribution, metabolism, and excretion (ADME) model to predict annual PFOA serum concentrations for each individual, and compared those predicted serum concentrations to observed 2005–2006 serum measurements.

Cross-sectional serum PFOA concentrations and questionnaire responses are available for 69,030 individuals who participated in the C8 Health Project in 2005–2006 (Frisbee et al. 2009; Steenland et al. 2009). The median 2005–2006 serum concentration for these individuals was 28.2 ppb (mean, 83.0 ppb). In this study, we included only the 48,998 participants who consented to share their residential address histories with the C8 Science Panel (2011). We identified 45,276 of these individuals who did not report DuPont as a past or present employer in their questionnaire responses; we assumed that these individuals did not have any significant occupational exposures and are the focus of our comparisons between predicted and observed PFOA serum concentrations.

The objective of our study was to reconstruct historical PFOA exposures and serum concentrations from 1951 to 2008 for participants in the C8 Health Project for use in a variety of epidemiologic analyses investigating whether past PFOA exposures beginning in 1951 are associated with historical health effects such as birth outcomes during the last few decades and whether cumulative exposures are associated with chronic diseases such as certain cancers in the eastern Ohio and western West Virginia region. Although 2005–2006 serum PFOA concentrations are available, these (and all biomarkers) primarily reflect current and recent exposures and may not be representative of exposures in earlier decades (Bartell et al. 2004; Paustenbach and Galbraith 2006).

## Materials and Methods

*Environmental fate and transport modeling.* We developed and integrated environmental fate and transport models to simulate PFOA concentrations in air, surface water, and groundwater. Each model component was linked to each other to model dispersion in air, percolation through soil with rainfall, mixing and transport with river water, and transport with groundwater flow. Because these environmental fate and transport processes occur in series, the output from each preceding model was used as the input for the next model. For example, the rainfall recharge from an unsaturated soil zone model and the river recharge from a surface water model were used as the input for groundwater flow and transport models

**Figure 1:**
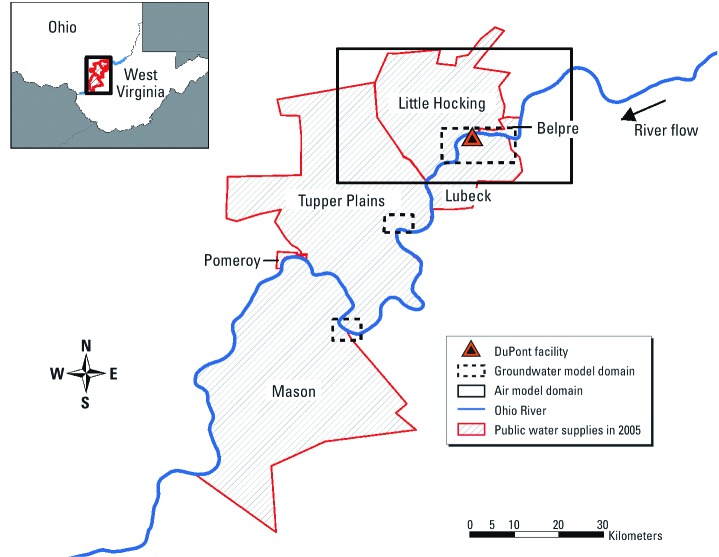
C8 Health Project study area, public water supply well locations, air model domain (black solid-line box), and groundwater model domain (black dashed-line boxes).

Air and groundwater model domains are shown in Figure 1. The groundwater model domains for the two downstream municipal water supply wells (Tuppers Plains and Mason County) are shown in smaller dashed-line boxes. These “domains” are the geographic regions for which historical air, soil, and water PFOA concentrations were estimated. Aerial deposition of PFOA is predicted to have been negligible at the contaminated public water systems outside this air model domain (Shin et al. 2011) as draw from the Ohio River is thought to have been the primary exposure pathway for those water systems. The environmental fate and transport models used to estimate PFOA air and water concentrations require dozens of parameters and detailed information regarding local meteorology and hydrogeology. Details of model optimization and calibration procedures were described previously (Shin et al. 2011). In that study (Shin et al. 2011), we estimated historical PFOA concentrations in air, groundwater, and six public water supplies within the model domains in Figure 1 for 1951–2008 by linking several environmental fate and transport modeling systems. The six public water districts were those covered by the 2005 legal settlement and whose residents participated in the 2005–2006 C8 Health Project (Figure 1): City of Belpre, Little Hocking Water Association, Tuppers Plains Chester Water District, and Village of Pomeroy water district, located in Ohio, and Lubeck and Mason public service districts, located in West Virginia.

*Exposure and dosimetry model.* There are several pathways for PFOA released from the DuPont plant to travel through the environment to reach the C8 Health Project participants (Shin et al. 2011). Although the most important route of exposure for most participants is thought to be ingestion of contaminated groundwater, inhalation of airborne particulates may also contribute to exposure. Thus, we considered inhalation and drinking water ingestion in our exposure and dosimetry model. We estimated average annual doses for individual pathways from the following dose equation:

*I_p_* = *C_E_* × *R*, [1]

where *I_p_* is the annual potential intake (micrograms per year), *C_E_* is the average annual exposure concentration (micrograms per liter for water, micrograms per cubic meter for air), and *R* is the uptake rate from the media (liters per year for water, cubic meters per year for air). We used many individual-specific determinants, including demographic information, residential histories, drinking water source at home and workplace, tap water consumption rates, and workplace histories, to estimate dose as described below.

*Residential histories and drinking water sources.* We mapped the distribution systems for the six water districts included in the exposure and dosimetry model using a geographic information system (GIS). Participants provided residential histories beginning in 1980 for locations that were geocoded by Battelle Memorial Institute (Columbus, Ohio) with ArcView (version 9.3; ESRI, Redlands, CA) using the ESRI StreetMap Premium North America NAVTEQ 2008 enhanced street data set as the reference address locator. Addresses were matched using a spelling sensitivity of 70 and a minimum match score of 65. Of the addresses with ZIP codes known to have contaminated water districts, 88% were successfully geocoded to the street level and the remaining were geocoded to the population-weighted ZIP code centroid. We spatially joined the geocoded addresses to the pipe distribution systems within GIS to determine if participants were serviced by one of the six contaminated public water districts. We confirmed that the geocoded street name matched the street serviced by the pipe. Participants also indicated in the residential histories whether their drinking water was from one of the six PFOA-contaminated public water districts, a public water district outside of the six, a private drinking water well, or unknown. We manually reviewed any discrepancies between the water district assignments and the self-reported water districts. This included *a*) addresses for which participants reported drinking water from one of the six PFOA-contaminated water districts but for which we determined they were not located on the distribution system based on their geocoded location (5%), and *b*) addresses that we determined should be serviced by one of the six PFOA-contaminated public water districts but for which participants self-reported being serviced by another (4%).

*Handling inconsistencies in residential histories.* Some residential histories were incomplete or contained records with overlapping time frames at two or more residential addresses. We handled overlapping times in the residential histories by averaging the predicted concentrations for each media during any years of overlap. Because the overall questionnaire, which included residential history, was partly designed to establish eligibility for participation in a lawsuit based on residence in the region surrounding the Washington Works facilities, we believe that gaps in residential history most likely reflect residence outside of that contaminated region. We therefore assumed that participants had no PFOA exposures above background levels during these gaps.

*Assignment of air and water exposure based on residential histories.* We used year-by-year residential locations to assign exposure concentrations. For air exposure, if one lived inside the air dispersion model domain (solid line box in Figure 1), we assigned the nearest concentrations from the grid model using the residential address geocode (*x*- and *y*-coordinates) or ZIP centroid if no residential address geocode was available. For individuals residing outside the grid model domain, we assigned no exposure for that residence. We assumed that indoor air exposure concentration was 0.1 times outdoor concentration because of the possible penetration and partial filtration and loss of PFOA into homes (Koponen et al. 2001). In addition, we assumed that the PFOA air concentration was 0 μg/m^3^ in vehicles and workplaces.

For drinking water exposure, if an individual lived within one of the six municipal water supplies, we assigned average predicted groundwater concentrations of each public well taken from layer 2 (middle layer) of the groundwater model, which consists of three layers depending on different geologic units and assigns all municipal pumping wells in layer 2, using the geocoded location of the individual public well. For years after granular activated carbon (GAC) treatment went online (starting in 2006–2008), we assigned zero drinking water exposure depending on the water district. For individuals with addresses inside the groundwater model domain (large black dashed-line box in Figure 1) who self-reported drinking water from a private well or who self-reported being serviced by a public water district other than the six public water supplies, we assigned the nearest concentrations to the shallowest layer 1 of the groundwater model defined by Shin et al. (2011) using the geocoded residential address, or ZIP centroid if the residential address could not be geocoded. If the residence is located inside the air dispersion model domain but outside the groundwater model domain, we assigned the concentration from the unsaturated soil zone model, assuming the soil type in the simulated zone to be predominant vertically from the ground surface to private wells. For individuals living outside both the air dispersion and groundwater model domains, we assigned zero drinking water exposure. If the water source was unknown, we assigned the weighted average concentrations of public and private wells. The weights were calculated by summing the pipe length for each public water distribution network and dividing by the total street length within the ZIP code. For a summary of exposure concentration assignments, see Supplemental Material, Table 1 (http://dx.doi.org/10.1289/ehp.1103729).

**Table 1:** Summary of serum predictions for different subgroups of participants.

Characteristic	*n*	*r*_s_	Median (ppb)		Underprediction*a*	Close approximation*a*	Overprediction*a*
			Predicted	Observed			
All participants	43,449	0.68	13.7	23.5	34.6	51.3	14.0
Water consumption data available	23,052	0.70	15.3	24.8	33.8	50.5	15.6
Residence in one of the six water districts in 2005/2006	23,971	0.75	27.7	36.2	24.2	58.8	17.0
Same residence and workplace in one of six water districts from 2001 to 2005	1,585	0.81	29.5	35.4	18.7	64.7	16.6
Same residence and workplace in one of six water districts from 2001 to 2005 and water consumption	1,103	0.82	33.2	38.6	16.7	65.4	18.0
Same residence and workplace not in one of six water districts from 2001 to 2005	3,095	0.32	5.4	15.0	56.6	36.3	7.0
Bottled-water drinkers	2,321	0.59	9.6	26.9	51.4	38.7	9.9
Nonvegetable growers	33,088	0.67	13.3	22.4	34.4	51.3	14.3
Vegetable growers	10,361	0.70	15.2	27.9	35.4	51.4	13.2
**a**Represents the percentage of model results within these categories.														

*Demographic information.* The C8 Health Project gathered self-reported (and parent-reported for children) demographic information, including race, age, sex, height, and weight. We obtained age- and sex-specific uptake rates including inhalation rate, drinking water ingestion rate, and activity time indoors and outdoors from the U.S. Environmental Protection Agency (EPA) *Exposure Factors Handbook* (U.S. EPA 2009), assuming they were applicable for 365 days per year. For adult exposure predictions, we applied self-reported body weights obtained at the time of survey throughout adulthood. For childhood exposure predictions, we assigned age- and sex-specific body weight from recommended values of the *Exposure Factors Handbook* (U.S. EPA 2009).

*Drinking water source.* Almost all participants (99%) reported their drinking water source as either public wells, private wells, or bottled water at the time of the serum sampling in 2005–2006. In addition, the bottled-water start year was available for bottled-water drinkers. About 5.3% of participants (*n* = 2,419) reported that they had bottled water as the primary drinking water source. However, for Little Hocking, self-reported bottled-water use (6.6%) was much lower than the proportion of households receiving free bottled water from the Little Hocking Water District at about the same time (81% of households by 30 December 2005) (Griffin 2008). Despite this discrepancy, we used self-reported bottled-water use information in the model because we could neither identify specific households participating in the bottled-water program nor assume that all members of a participating household routinely consumed bottled water. For the self-reported bottled-water drinkers, we assumed no PFOA exposure contribution from residential water consumption after they reportedly started drinking bottled water.

*Actual tap water consumption at home.* Approximately 50% of participants (*n* = 24,450) provided their best estimates of the total number of cups per day, including plain tap water that they drank or used to make their own hot or cold beverages such as coffee, tea, drinks using water-flavoring additives, and juice from concentrate before it became known that PFOA had contaminated the water. The volume of one cup is about 240 mL. The average self-reported tap water consumption amount per day is 1.37 L for these 24,450 participants, a value remarkably close to the U.S. EPA default value of 1.40 L (U.S. EPA 2009). We used self-reported tap water consumption data in our exposure model when available and assumed the U.S. EPA water consumption value for participants with unknown tap water consumption rates. We assumed that any individual’s water consumption rate was constant over the entire exposure period.

*Water consumption at work.* Approximately 80% of participants (*n* = 36,226) provided some work history, including self-reported employment start and end years, the water district serving each workplace, and workplace ZIP code. Most of those without any reported work history were children, students from elementary school to college, and adult women; we assumed that most of these individuals had little or no work history to report. We computed drinking water ingestion exposure from workplaces using the same method applied in residential histories. However, we assumed that all participants who self-reported work histories consumed public well water at the workplace with median self-reported tap water consumption amount (1.37 L) because relative contributions of different drinking water sources (public wells, private wells, bottled water) and actual tap water consumption rate from the workplace were not available in the questionnaire.

We compared predicted and observed PFOA median serum concentrations of individuals who worked in one of the six municipal water districts from 2001 to 2005 but lived outside of the six water districts. For those who have residential histories in the water districts with low PFOA water concentrations (e.g., Pomeroy or Mason County or outside the six water districts) and workplace histories in the water districts with high PFOA water concentrations (e.g., Little Hocking or Lubeck), predicted serum concentrations were lower than observed serum concentrations when we assumed that 100% of water was ingested at home, suggesting some unaccounted water consumption at the workplace. Therefore, we tried several different ratios of home-to-workplace water consumption in the model; 70% of residential and 30% of workplace water consumption resulted in the highest correlation coefficient between predicted and observed serum concentration, and this was used in the final model.

*ADME model.* We assumed that serum PFOA concentrations of participants in the C8 Health Project were contributed from both the emissions by the Washington Works Plant and background exposures not originating from air and water emissions from that facility, for example, PFOA consumer products such as food packaging, nonstick cooking material, and stain-resistant upholstery and carpeting. Little information is available regarding historical background serum concentrations other than NHANES median serum concentrations of 5.2 μg/L analyzed during 1999–2000 and 3.9 μg/L during 2003–2004 (Calafat et al. 2007). Assuming that background serum concentration in 1950 was 0 μg/L, we interpolated linearly year-specific background serum concentration using three data time points or periods including *a*) 1950, *b*) 1999–2000, and *c*) 2003–2004. To consider the contribution from the Washington Works plant for serum concentrations, we developed an ADME model to estimate the amount of PFOA reaching and remaining in blood. We used the following single-compartment ADME model (Equation 2c, below) to estimate serum concentrations for each year with assumptions of piecewise-constant exposure rate and first-order excretion (Bartell 2003). Because the volume of distribution changes more rapidly during childhood, we applied a PFOA mass-basis step function and divided PFOA mass by age- and sex-specific volume of distribution to compute serum concentrations:

*C_t_* = *C_t_*_,ww_ + *C_t_*_,bc_, [2a]

*C_t_*_,bc_ = β_1_ × (*t* – 1950) if *t* < 1999, *C_t_*_,bc_ = *C*_2000,bc_ + β_2_ × (*t* – 1999)  if 1999 ≤ *t* ≤ 2004, [2b]

*C_t_*_,bc_ = β_1_ × (*t* – 1950) if *t* < 1999, *M_t_*_,ww_ = *M_t_*_–1,ww_ × *e*^–^*^k^* + (1 – *e*^–^*^k^*) × (*I_t_* / *k*), [2c]

*C_t_*_,ww_ = *M_t_*_,ww_ / *V*, [2d]

where *C_t_* is the serum PFOA concentration (micrograms per liter) contributed from background concentration and the Washington Works emissions for year *t*, *C_t,_*_bc_ is background serum PFOA concentration (micrograms per liter) for year *t*, *C_t_*_,ww_ is the serum PFOA concentration (micrograms per liter) due to the emissions from the Washington Works for year *t*, β_1_ is 0.11 [(C_1999_ – C_1950_) ÷ (1999 – 1950)] (micrograms per liter per year), β_2_ is –0.33 [(C_2004_ – C_2000_) ÷ (2004– 2000)] (micrograms per liter per year), *M_t_*_,ww_ is the serum PFOA mass (micrograms) due to the emissions from the Washington Works for year *t*, Δ*t* is 1 year, *I_t_* is the total mass of PFOA ingested (micrograms per year) for year *t*, *V* is age- and sex-specific volume of distribution (liters), and *k* is an excretion rate coefficient for PFOA (per year).

For participants whose body weight was available at the time of serum sampling event, we multiplied the recommended volume of distribution per weight, 0.181 L/kg for males and 0.198 L/kg for females (Butenhoff et al. 2004), by self-reported body weight. For participants without a reported body weight, we multiplied the recommended volume of distribution per weight by median age- and sex-specific body weights recommended from the *Exposure Factors Handbook* (U.S. EPA 2009). For the excretion rate coefficient, we used a half-life of 3.5 years from a study with 5 years of follow-up (Olsen et al. 2007).

*Perinatal exposure.* We also considered perinatal exposure transplacentally or via breast-feeding because the human fetus could be exposed to PFOA transferred from mother’s blood across the placental barrier or during breast-feeding. For example, Fromme et al. (2010) reported that for a randomly selected population in Munich, Germany, PFOA cord serum values (*n* = 33) ranged from 0.5 to 4.2 ppb and that cord blood PFOA concentration was 58% of maternal blood concentrations (*R*^2^ = 0.83). These results indicate that the human fetus is exposed to PFOA transferred from mother’s blood across the placental barrier. We estimated the weighted average of the cord blood:maternal PFOA serum ratio for newborns using reported PFOA ratios from seven published studies (Fei et al. 2007; Fromme et al. 2010; Hanssen et al. 2010; Kim et al. 2011; Midasch et al. 2007; Monroy et al. 2008; Needham et al. 2011) to obtain the value 0.785 for use in our model [see Supplemental Material, Table 2 (http://dx.doi.org/10.1289/ehp.1103729)]. Although we did not have cord blood data, we were able to estimate the median of 1-year-old infant as maternal PFOA serum ratio using serum from 40 infant–mother pairs matched among our participants, and we obtained the value 1.27. Thus, we assumed that serum concentrations for newborns and 1-year-old infants were 78.5% and 127% of their mother’s predicted serum concentration, respectively.

## Results

**Figure 2:**
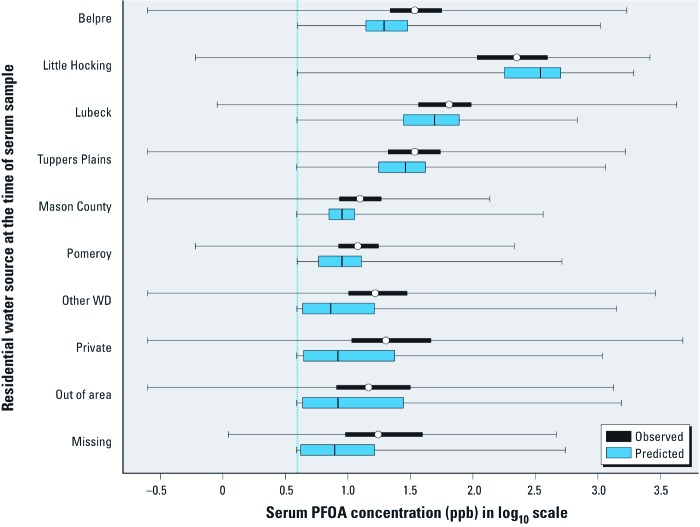
Box-and-whisker plots of predicted and observed serum PFOA concentrations (parts per billion) in 2005–2006 by water district (WD). The vertical line indicates the median background serum concentration in 2005–2006 in human serum samples. Lines and circles within boxes indicate median concentrations, boxes correspond to the 25th and 75th percentiles, and whiskers indicate minimum and maximum values.

Figure 2 shows box-and-whisker plots of predicted and observed serum PFOA concentrations (parts per billion) in log_10_ scale by water districts during the sampling period of 2005–2006. Participants who reported living in Little Hocking in 2005–2006 had the highest median serum concentrations. Of the six public water supplies, median serum concentrations were observed and predicted for Little Hocking, Lubeck, Tuppers Plains, Belpre, Mason County, and Pomeroy. All predicted medians are aligned with observed medians within 0.5 orders of magnitude. For those residing outside the six qualifying water districts, serviced by private wells, or missing a water district assignment at the time of serum sample, predictions appear less reliable, based on the wide ranges of predictions and the distances between predicted medians and observed medians. Serum concentrations for Little Hocking participants were likely overpredicted because of underreported bottled-water consumption, as discussed in “Materials and Methods.” Although predicted and observed maximum serum concentrations are of the same magnitude with the exception of Lubeck, predicted minimum concentrations are always larger than observed minimum concentration because of the default assignment of background concentration of 3.9 ppb for all individuals in 2005.

Summary statistics for different subsets of participants are described in Table 1. We included only participants with observed serum concentrations and without reported employment history by DuPont in the summary statistics. The Spearman’s rank correlation coefficient for predicted versus observed serum concentrations is 0.67. Predicted and observed median serum concentrations are 14.2 and 24.3 ppb, respectively. Predicted water concentrations from our linked modeling systems provide more credible drinking water exposure concentrations for public water systems in the six municipal water districts compared with other public and private water sources for which geocoded well locations or well depths were not available. The Spearman’s rank correlation coefficients for predicted versus observed serum samples increased with more accurate water consumption and water concentration information, albeit not greatly. For example, if restricted to those with a self-reported water consumption rate, the correlation coefficient was increased from 0.67 to 0.69. For people who had both lived and worked in one of six public water districts for 5 consecutive years before the sampling event and provided water consumption information, the correlation coefficient was increased from 0.67 to 0.82. On the other hand, for those who had not lived and worked in one of the six public water districts for 5 consecutive years before the sampling event, the correlation coefficient was decreased from 0.67 to 0.32. We also provide two log–log scatter plots using a random sample of 1,000 participants drawn from all participants [see Supplemental Material, Figure 2 (http://dx.doi.org/10.1289/ehp.1103729)], and using participants (*n* = 1,074) who had the same residence and workplace water district from 2001 to 2005 and provided water consumption information (see Supplemental Material, Figure 3.) Comparing the correlation coefficients and slopes of these two figures, predicted serum concentrations are more correlated with observed serum concentrations when we have higher quality exposure concentrations (i.e., six qualifying water districts) and individual-specific water consumption rates based on the correlation coefficients (0.67 vs. 0.82) and slopes (0.78 vs. 0.89).

We also assessed the performance of the exposure and the ADME model by categorizing modeled results as overprediction, underprediction, and close approximation compared with serum measurements in 2005–2006. Overprediction is defined as modeling values greater than 2 times the observed sampling data, underprediction as the values less than 0.5 times the observed sampling data, and close approximation as the values between 0.5 and 2 times the observed sampling data. Similar to the effect on the correlation coefficient, the percentage of close approximations increased with more accurate water consumption and water concentration information (Table 1).

We used the half-life of 2.3 years estimated using a subpopulation of 200 participants from the C8 Health Project (Bartell et al. 2010) in an alternative analysis, to compare the model performance with different elimination rates. The Spearman’s rank correlation coefficient between predicted and observed concentrations (*r*_s_ = 0.68) is similar to the result with a half-life of 3.5 years, but the predicted median with a 2.3 year half-life (9.3 ppb) is farther from the observed median (24.3 ppb) than the predicted median with a 3.5 year half-life (14.2 ppb).

We conducted additional analyses to determine whether the inclusion of maternal exposure transplacentally or via breast-feeding improves the prediction of children’s serum concentrations. We excluded Little Hocking children in these comparisons because of the bottled-water distribution program and the post-2000 awareness of Little Hocking PFOA tap water contamination that may have affected parental use of tap water in formula and food preparation for infants. A summary of the contribution of maternal transfer to children by specific age range is shown in Supplemental Material, Table 3 (http://dx.doi.org/10.1289/ehp.1103729). Inclusion of maternal exposure for newborns and 1-year-old infants improved the correlation coefficients and prediction of serum concentrations. Overall correlation coefficients for children 1–9 years of age at time of serum measurement in 2005 increased from 0.52 to 0.61 after incorporating maternal transfer. Predicted medians for models including a maternal transfer component were closer to observed medians compared with those without modeling the maternal contribution to infant serum PFOA.

## Discussion

This study is unique in epidemiology and environmental health science in that about 49,000 individuals provided blood samples and answered a questionnaire about residential history and water use. Reconstructing individualized retrospective exposure estimates for this population is a key step in determining if there is an association between historical PFOA exposure and adverse health effects among community residents. The challenge of this study was in estimating environmental exposures that could occur through multiple pathways. Although cumulative exposure over the lifetime is dominated by water ingestion for nearly all participants, annual exposures are more complicated. For those who lived in areas with air contamination due to emissions from the plant (primarily served by Little Hocking and Belpre), air inhalation contributed more than water ingestion to annual PFOA exposure early in the history of emissions from the plant because of the retardation of PFOA movement through the unsaturated and saturated zones before reaching public and private wells. Drinking water ingestion from contaminated groundwater began to be the dominant route for annual PFOA exposures by about 1974 in Little Hocking and by about 1990 in Belpre [see Supplemental Material, Figure 4 (http://dx.doi.org/10.1289/ehp.1103729)]. However, the biggest challenge was to determine intake rates for drinking water ingestion because of a lack of activity patterns, including historical actual tap water consumption at home or work.

Observed serum concentrations in Little Hocking Water Association are likely lower than predicted values because of a popular bottled-water distribution/reimbursement program that started in August 2005. Some participants may have also begun using home GAC filtration or purchased their own bottled water years before serum sampling, particularly after local news coverage on the water supply contamination raised public awareness starting in 2001. GAC filter use at home was not ascertained for this population. We asked participants if they consumed bottled water in 2005–2006, but this behavior likely changed dramatically over time and could have been substantially underreported if C8 Health Project participants believed that their answers could affect the compensation they would eventually receive from a legal settlement. Indeed, only 3,728 of 69,030 participants in the C8 Health Project reported bottled-water consumption (Steenland et al. 2009). In contrast, > 9,600 Little Hocking Water Association customers (many of whom were purchasing water for several people in a household) were participating in its emergency bottled-water program by 2007 (Griffin 2008).

Several studies had implicated local vegetable consumption as an exposure source in this region, including the C8 Health Project (Bartell et al. 2010; Emmett et al. 2006; Steenland et al. 2009). About 23% of participants reported that they had grown their own vegetables before the survey. The median and mean differences between those reporting growing their own vegetables and those not growing their own vegetables were 6 ppb and 27 ppb, respectively. However, because few individual-specific data are available regarding this exposure route in the C8 Health Project participants, we did not include this route in the exposure model.

Our year-by-year exposure and serum estimates are being used in ongoing epidemiologic studies of past and recent health outcomes. More effort and resources to characterize parameters and inputs could improve model predictions and decrease prediction uncertainty. In particular, we expect that individual-specific information on drinking water consumption rates and nonresidential water consumption patterns might substantially improve our individual predictions. Individual-level data on consumption of local and homegrown produce could also improve these model predictions, particularly for current and future exposures in this region. It is unusual to have such extensive biomonitoring data available to compare with individual-level exposure reconstructions. These data helped confirm that our exposure estimates are reasonable and also demonstrate the potential value of obtaining high-quality residential histories and water district assignments for waterborne exposures. These findings may also help epidemiologists appraise uncertainty in exposure–disease associations due to exposure mismeasurement, although serum measurements primarily reflect recent exposures and should not be viewed as a gold standard for historical exposure reconstruction.

## Supplemental Material

(295 KB) PDF
