# Erratum: Perfluorooctanoic Acid Concentrations for Participants in the C8 Health Project [119(12):1760–1765 (2011)]

**DOI:** 10.1289/ehp.1306804

**Published:** 2013-04-01

**Authors:** Hyeong-Moo Shin, Verónica M. Vieira, P. Barry Ryan, Kyle Steenland, Scott M. Bartell

**Affiliations:** 1 Department of Public Health Sciences, University of California, Davis, Davis, California, E-mail: hmshin@ucdavis.edu; 2 Department of Environmental Health, Boston University, Boston, Massachusetts; 3 Department of Environmental Health, Emory University, Atlanta, Georgia; 4 Program in Public Health, Department of Statistics, University of California, Irvine, Irvine, California

In “Retrospective Exposure Estimation and Predicted versus Observed Serum Perfluorooctanoic Acid Concentrations for Participants in the C8 Health Project” (Shin et al. 2011b), we reported estimates of historical perfluoro-octanoic acid (PFOA) exposures and serum concentrations for 45,276 non-occupationally exposed partici-pants in the C8 Health Project who consented to share their residential histories. We recently discovered an inconsistency in our estimates of historical water concentrations for some of these participants. For four public water districts (Belpre, Little Hocking, New Lubeck, and Tuppers Plains), the water concentrations used to estimate exposures and predicted serum concentrations were not consistent with water concentration estimates we reported in another article, “Environmental Fate and Transport Modeling for Perfluorooctanoic Acid Emitted from the Washington Works Facility in West Virginia” (Shin et al. 2011a). The difference in water concentration estimates slightly affects our estimates of PFOA exposures and serum concentrations. The Spearman’s rank correlation coefficient (*r*_S_) between updated serum estimates and the originally reported values is 0.996.

**Table 1:** Summary of serum predictions for different subgroups of participants.

Characteristic	*n*	*r*_s_	Median (ppb)		Underprediction*a*	Close approximation*a*	Overprediction*a*
			Predicted	Observed			
All participants	43,449	0.68	13.7	23.5	34.6	51.3	14.0
Water consumption data available	23,052	0.70	15.3	24.8	33.8	50.5	15.6
Residence in one of the six water districts in 2005/2006	23,971	0.75	27.7	36.2	24.2	58.8	17.0
Same residence and workplace in one of six water districts from 2001 to 2005	1,585	0.81	29.5	35.4	18.7	64.7	16.6
Same residence and workplace in one of six water districts from 2001 to 2005 and water consumption	1,103	0.82	33.2	38.6	16.7	65.4	18.0
Same residence and workplace not in one of six water districts from 2001 to 2005	3,095	0.32	5.4	15.0	56.6	36.3	7.0
Bottled-water drinkers	2,321	0.59	9.6	26.9	51.4	38.7	9.9
Nonvegetable growers	33,088	0.67	13.3	22.4	34.4	51.3	14.3
Vegetable growers	10,361	0.70	15.2	27.9	35.4	51.4	13.2
**a**Represents the percentage of model results within these categories.														

A cohort follow-up also resulted in the addition of 118 new partici-pants (all who had provided consent), as well as the removal of 1,945 participants who had been newly identified as having historical occupational exposure to PFOA. Updated summary statistics comparing predicted and observed serum concentrations are shown in Table 1. Among all participants (*n* = 43,449), updated serum PFOA concentration predictions are largely similar to the originally reported values (e.g., medians of 13.7 ppb and 14.2 ppb for updated and originally reported values, respectively; for predicted versus observed serum concentrations, *r*_S_ = 0.677 and 0.674 for updated and originally reported values, respectively). Updating the water concentrations resulted in a decrease of 0.2 ppb in median serum concentration estimates, and the removal of newly identified DuPont workers resulted in a decrease of 0.3 ppb. We found no noticeable change in the updated summary statistics from the addition of the 118 new participants.

In summary, the two sets of predictions are very similar and match the observed serum equally well. This update does not substantially change the conclusions of our study (Shin et al. 2011b).
