# Supplementary material for: Retrospective Exposure Estimation and Predicted versus Observed Serum Perfluorooctanoic Acid Concentrations for Participants in the C8 Health Project
Source: Environ Health Perspect. 2011 Aug 3;119(12):1760–5. doi: 10.1289/ehp.1103729 (PMC3261988; doi:10.1289/ehp.1103729)
Supplement: (295 KB) PDF [file ehp.1103729.s001.pdf]

Supplemental Material

Retrospective Exposure Estimation and Predicted versus Observed Serum Perfluorooctanoic Acid Concentrations for Participants in the C8 Health Project

Hyeong-Moo Shin[1], Verónica M. Vieira[2], P. Barry Ryan[3], Kyle Steenland[3, 4], and Scott M. Bartell[5]

[1]School of Social Ecology, University of California, Irvine, California, USA

[2]Department of Environmental Health, Boston University, Boston, Massachusetts, USA

[3]Department of Environmental Health, Emory University, Atlanta, Georgia, USA

[4]C8 Science Panel (http://www.c8sciencepanel.org)

[5]Program in Public Health, Department of Statistics, and Department of Epidemiology, University of California, Irvine, California, USA

TABLE OF CONTENTS

Tables & Figures **3**

Supplemental Material, Table 1. Summary of exposure concentrations assignment by data type 3

Supplemental Material, Table 2. The cord: maternal PFOA ratio in blood based on literature review 4

Supplemental Material, Figure 2. A log-log plot of predicted vs. observed concentrations from a random sample of 1000 participants drawn from all participants. Linear trends are shown as a solid line and predicted and observed concentrations are shown as points. 5

Supplemental Material, Figure 3. A log-log plot of predicted vs. observed concentrations for participants (n =1,074) who had same residence & workplace in 1 of 6 water districts from 2001 to 2005 and provided water consumption information. Linear trends are shown as a solid line and predicted and observed concentrations are shown as points. 6

Supplemental Material, Table 3. Summary of contribution of maternal transfer to children by specific age range excluding Little Hocking children 7

Supplemental Material, Figure 4. Relative contribution of air inhalation exposure to total exposure from the Washington Works facility (%) as a function of time for each water district. Exposure was averaged based on historical water district 8

References **9**

TABLES & FIGURES

Supplemental Material, Table 1. Summary of exposure concentrations assignment by data type

WD [a] code	Data Type (n)	Geographic Location	Number of Records	Air conc.	Water source
1-6 (n=65,470)	Geocode +ZIP (n=64,679)	Inside Air & Inside GW [b]	22,689	X, Y	Public
		Inside Air & Outside GW	16,706		
		Outside Air & Outside GW	25,284	0	
	No Geocode+ ZIP (n=791)	Inside Air & Inside GW	12	X, Y (ZIP centroid)	Public
		Inside Air & Outside GW	148		
		Outside Air & Outside GW	631	0	
7,8,0 (n=85,154)	Geocode +ZIP (n=78,992)	Inside Air & Inside GW	1,181	X, Y	Private
		Inside Air & Outside GW	23,161		Private [c]
		Outside Air & Outside GW	54,650	0	0
	No Geocode+ ZIP (n=5,082)	Inside Air & Inside GW	65	X, Y (ZIP centroid)	Private
		Inside Air & Outside GW	448		Private [c]
		Outside Air & Outside GW	4,569	0	0
	No Geocode+ No ZIP (n=1,080)	NA	1,080	0	0
blank (n=1,247)	Geocode +ZIP (n=1,174)	Inside Air & Inside GW	97	X, Y	Mix (Public, Private)
		Inside Air & Outside GW	235		Mix (Public, Private [c])
		Outside Air & Outside GW	842	0	Mix (Public, Private=0)
	No Geocode+ ZIP (n=73)	Inside Air & Inside GW	0	X, Y (ZIP centroid)	Mix (Public, Private)
		Inside Air & Outside GW	33		Mix (Public, Private [c])
		Outside Air & Outside GW	40	0	Mix (Public, Private=0)
Total	151,871		151,871		

[a] Water district code reported in the study

[b] Groundwater model domain

[c] Private well concentrations from the vadose zone model for outside groundwater model domain

Supplemental Material, Table 2. The cord: maternal PFOA ratio in blood based on literature review

Reference	Sample size	PFOA ratio	Method of estimating the PFOA ratio
Fromme et al. (2010)	27	0.745	calculated using the provided regression equation
Kim et al. (2011)	20	0.694	calculated using the provided regression equation
Midasch et al. (2007)	11	1.260	provided mean of ratio
Fei et al. (2007)	50	0.680	calculated from provided mean ratio of maternal:cord
Needham et al. (2010)	12	0.720	provided median of ratio
Monroy et al. (2008)	101	0.866	calculated ratio of provided mean
Hanssen et al. (2020)	58	0.710	calculated from provided mean ratio of maternal:cord

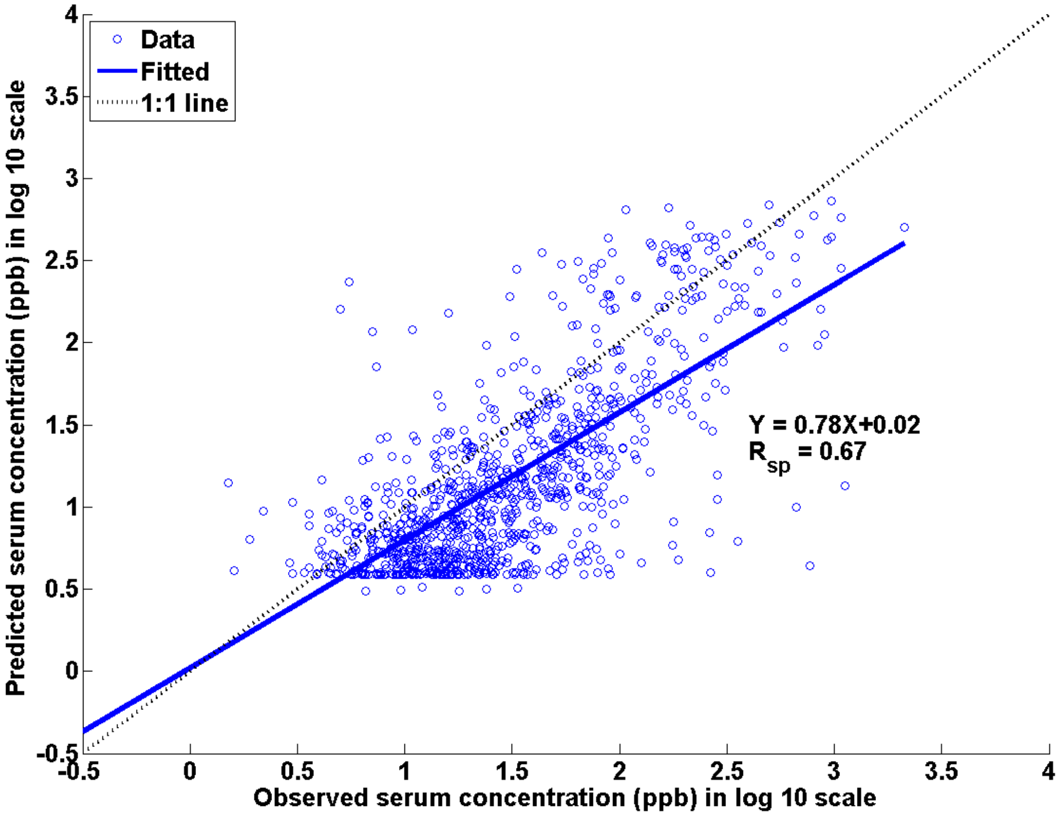

Supplemental Material, Figure 2. A log-log plot of predicted vs. observed concentrations from a random sample of 1000 participants drawn from all participants. Linear trends are shown as a solid line and predicted and observed concentrations are shown as points.

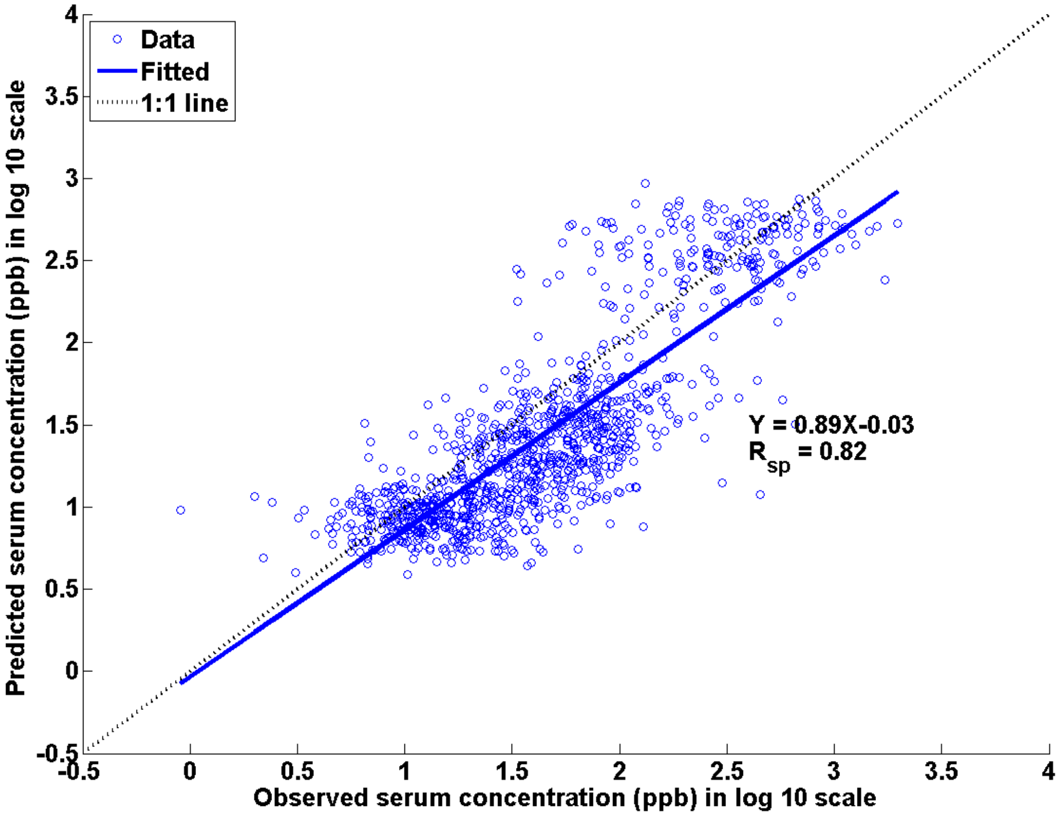

Supplemental Material, Figure 3. A log-log plot of predicted vs. observed concentrations for participants (n =1,074) who had same residence & workplace in 1 of 6 water districts from 2001 to 2005 and provided water consumption information. Linear trends are shown as a solid line and predicted and observed concentrations are shown as points.

Supplemental Material, Table 3. Summary of contribution of maternal transfer to children by specific age range excluding Little Hocking children

Source		No maternal transfer		Maternal transfer		
Age	N	R_{sp}	Predicted median (ppb)	R_{sp}	Predicted median (ppb)	Observed median
1-2	7	0.00	8.7	0.61	33.9	19.0
2-3	93	0.32	7.6	0.66	30.0	27.3
3-4	136	0.44	15.2	0.50	34.4	21.1
4-5	198	0.60	18.2	0.58	34.0	21.3
5-6	213	0.50	19.8	0.68	28.3	24.9
6-7	281	0.58	20.3	0.62	28.5	24.4
7-8	336	0.59	21.1	0.65	28.0	23.6
8-9	376	0.46	17.1	0.55	21.0	19.0
Total	1692	0.52	17.8	0.61	28.5	22.7

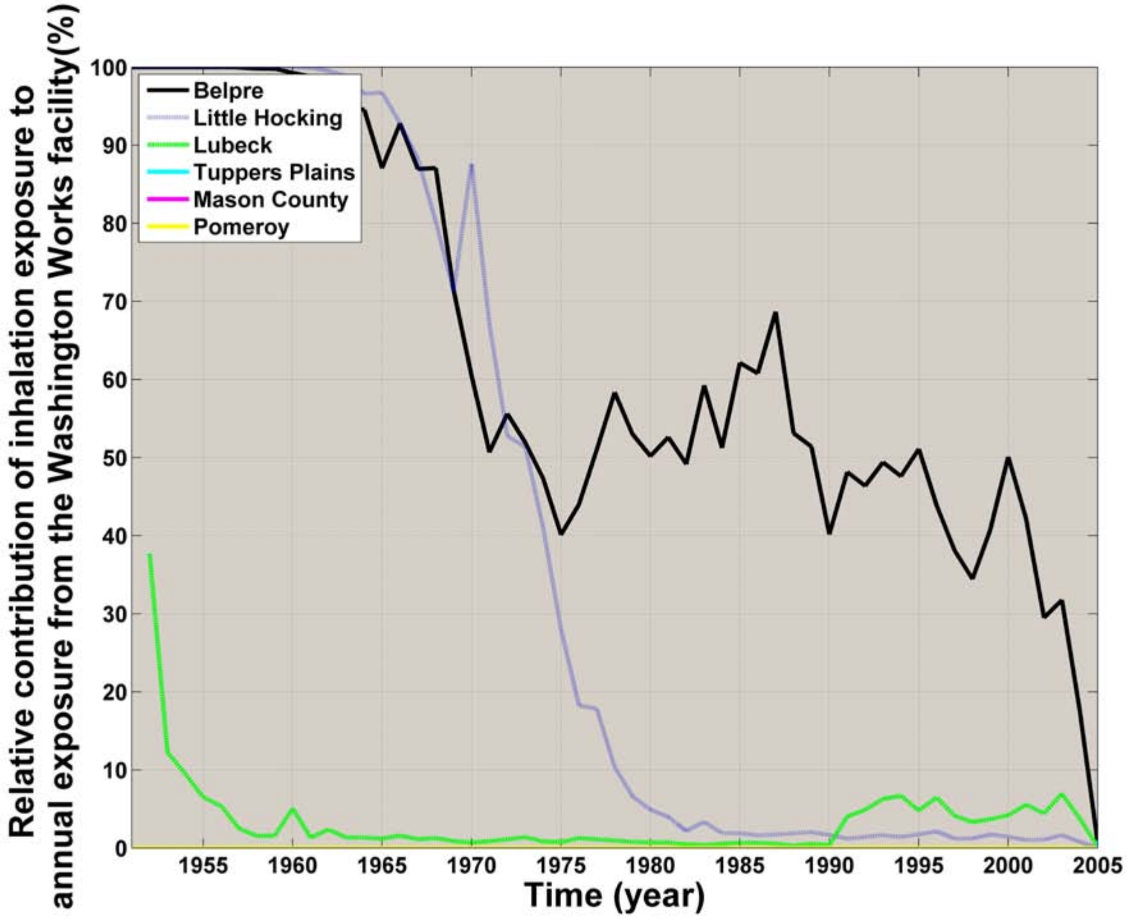

Supplemental Material, Figure 4. Relative contribution of air inhalation exposure to annual PFOA exposure from the Washington Works facility (%) as a function of time for each water district. Exposure was averaged based on historical water district

REFERENCES

Fei CY, McLaughlin JK, Tarone RE, Olsen J. 2007. Perfluorinated chemicals and fetal growth: A study within the Danish National Birth Cohort. Environ Health Perspect 115:1677–1682.

Fromme H, Mosch C, Morovitz M, Alba-Alejandre I, Boehmer S, Kiranoglu M, et al. 2010. Pre- and Postnatal Exposure to Perfluorinated Compounds (PFCs). Environ Sci Technol 44:7123–7129.

Hanssen L, Roellin H, Odland JO, Moe MK, Sandanger TM. 2010. Perfluorinated compounds in maternal serum and cord blood from selected areas of South Africa: results of a pilot study. J Environ Monitor 12(6): 1355–1361.

Kim SK, Lee KT, Kang CS, Tao L, Kannan K, Kim KR, et al. 2011. Distribution of perfluorochemicals between sera and milk from the same mothers and implications for prenatal and postnatal exposures. Environ Pollut 159(1): 169–174.

Midasch O, Drexler H, Hart N, Beckmann MW, Angerer J. 2007. Transplacental exposure of neonates to perfluorooctanesulfonate and perfluorooctanoate: a pilot study. Int Arch Occ Env Hea 80(7):643–648.

Monroy R, Morrison K, Teo K, Atkinson S, Kubwabo C, Stewart B, et al. 2008. Serum levels of perfluoroalkyl compounds in human maternal and umbilical cord blood samples. Environ Res 108(1):56–62.

Needham LL, Gandjean P, Heinzow B, Jorgensen PL, Nielsen F, Patterson DG, Sjodin A, Turner WE, Weihe P. 2011. Partition of environmental chemicals between maternal and fetal blood and tissues. Environ Sci Technol 45:1121-1126.
